# Impact of Inflammation on Myeloproliferative Neoplasm Symptom Development

**DOI:** 10.1155/2015/284706

**Published:** 2015-10-11

**Authors:** Holly L. Geyer, Amylou C. Dueck, Robyn M. Scherber, Ruben A. Mesa

**Affiliations:** ^1^Division of Hospital Internal Medicine, Mayo Clinic, Scottsdale, AZ 85054, USA; ^2^Department of Statistics, Mayo Clinic, Scottsdale, AZ 85054, USA; ^3^Division of Hematology and Medical Oncology, Mayo Clinic, Scottsdale, AZ 85054, USA

## Abstract

Myeloproliferative neoplasms (essential thrombocythemia, ET; polycythemia vera, PV; myelofibrosis, MF) are monoclonal malignancies associated with genomic instability, dysregulated signaling pathways, and subsequent overproduction of inflammatory markers. Acknowledged for their debilitating symptom profiles, recent investigations have aimed to determine the identity of these markers, the upstream sources stimulating their development, their prevalence within the MPN population, and the role they play in symptom development. Creation of dedicated Patient Reported Outcome (PRO) tools, in combination with expanded access to cytokine analysis technology, has resulted in a surge of investigations evaluating the potential associations between symptoms and inflammation. Emerging data demonstrates clear relationships between individual MPN symptoms (fatigue, abdominal complaints, microvascular symptoms, and constitutional symptoms) and cytokines, particularly IL-1, IL-6, IL-8, and TNF-*α*. Information is also compiling on the role symptoms paradoxically play in the development of cytokines, as in the case of fatigue-driven sedentary lifestyles. In this paper, we explore the symptoms inherent to the MPN disorders and the potential role inflammation plays in their development.

## 1. Introduction

In 1951, Sir William Dameshek postulated the concept of “myeloproliferative disorders” and furthermore ascribed their development to “a hitherto of undiscovered stimulus.” The past half-century has since brought light to the cryptic “stimulus” believed to drive these disabling neoplasms. Developing from a host of myelostimulatory mutations, myeloproliferative neoplasms (MPNs) including polycythemia vera (PV), essential thrombocythemia (ET), and myelofibrosis (MF) propagate through an evolving cascade of inflammatory conduits well documented to inflict dramatic symptomatology and impair quality of life. Great gains have been made in our understanding of how these disrupted signaling pathways coalesce into dysregulated synthesis of cytokines, chemokines, and reactive species that ultimately induce symptoms. In this paper, we discuss the role inflammation plays in MPN pathobiology, disease advancement, and symptom development.

## 2. Characterization of MPN Symptoms

The burdensome symptom profile is arguably the most recognizable feature of the MPN disease process and in itself may contribute to reduced life expectancy, as observed in myelofibrosis risk scoring [1, 2]. Prominent symptoms include fatigue (92.7%), early satiety (61.9%), abdominal pain (45.9%), abdominal discomfort (53.2%), inactivity (60.5%), headache (48.3%), concentration problems (61.7%), dizziness (55.2%), numbness (61.3%), insomnia (65.4%), sad mood (62.7%), sexuality problems (57.9%), cough (46.4%), night sweats (56.4%), itching (52.6%), bone pain (48.5%), fever (20.2%), weight loss (34.2%), and impaired quality of life (84.2%) [3]. The MPN symptom burden has been closely examined for its impact on patient daily living through the MPN LANDMARK survey. This study systematically surveyed 813 MPN patients and discovered that MPN symptoms negatively impacted work hours, number of sick days taken, the need for medical disability and/or early retirement, and overall activities of daily living. Patients additionally described feeling anxious and worried about their conditions (MF, 91%; PV, 78%; ET, 74%) which in turn compromised overall quality of life (MF, 81%; PV, 66%; ET, 57%) [4]. Adding to the complexity is the recent revelation that MPN symptoms indeed promote the development of other symptoms. An investigation of the symptom of insomnia revealed that the complaint correlates closely with most other MPN related symptoms and functional domains [5]. A similar study investigating correlations with MPN-related sexuality complaints found that this symptom also correlated with other MPN symptoms (insomnia, depression/sad mood, night sweats, and QOL), as well as emotional, cognitive, and social domains of functioning [6].

It has been well recognized that the prevalence and severity of symptoms differ by MPN subtype. However, more recent studies have demonstrated that significant heterogeneity exists even within MPN subtypes. A prospective evaluation of 1470 MPN patients discovered the presence of five clusters in PV and ET, respectively, and four clusters in MF [7]. Symptom clusters in ET and PV differed by clinical variables including age, language, gender, the presence of laboratory abnormalities, spleen size, history of hemorrhage, and Myeloproliferative Neoplasm Symptom Assessment Form Total Symptom Score (MPN-SAF TSS) value. Notably, neither PV nor ET clusters differed by risk scores suggesting symptomatology likely presents independent of disease stage and risk scoring tools should not be applied as surrogate measurements of disease severity. In MF, clusters differed by a variety of clinical variables as well as risk scores (DIPSS) with increasing degrees of symptomatology correlating with higher risk score categories.

Recent efforts have aimed to analyze the scope and extent of MPN symptoms in a systematic format. The first investigation was completed in 2007 as a self-reported internet survey of 1179 MPN patients [8]. This revealing study showed that fatigue, pruritus, bone pain, fevers, and weight loss led to restricted participation in physical and social functions and furthermore that available treatment regimens including androgens, steroids, hydroxyurea, and erythropoiesis-stimulating agents not only failed to improve the symptom burden but paradoxically contributed to its development. This survey served as a benchmark for the development of three MPN-specific PRO tools: MF-SAF, MPN-SAF, and MPN-SAF TSS/MPN-10. From these instruments, we have gathered key information on both the spectrum and severity of MPN symptoms. Below we discuss these tools individually (Table 1).

### 2.1. MF-SAF

The Myelofibrosis Symptom Assessment Form (MF-SAF) was created in 2009 and served as the first validated MPN Patient Reported Outcome (PRO) tool to be made available for clinical and trial settings [9]. A 20-item instrument, the survey attempted to capture the most common symptoms within myelofibrosis and content included issues related to catabolic/proliferative symptoms, quality of life, fatigue, and splenomegaly-associated issues. Questions were constructed in a “yes,” “no,” or 0 (absent) to 10 (worst imaginable) scale. The tool proved useful in the open label phase II trial of the* JAK2* inhibitor, ruxolitinib [10].

### 2.2. MPN-SAF

The Myeloproliferative Neoplasm Symptom Assessment Form (MPN-SAF) was developed two years later in efforts to capture the symptoms within PV and ET as well as MF [3]. This survey included the items present within the MF-SAF, along with questions related to microvasculature complications such as headaches, concentration problems, lightheadedness, dizziness, vertigo, numbness/tingling, and sexual dysfunction. This expanded version was structured in a similar format to the MF-SAF and proved beneficial in evaluation of a variety of novel targeted compounds including ruxolitinib, LY2784544, SAR302503, Vorinostat, and pegylated interferon [11–13].

### 2.3. MPN-SAF TSS (MPN-10)

The Myeloproliferative Neoplasm Symptom Assessment Form Total Symptom Score (MPN-SAF TSS; MPN-10) is an abbreviated version of the MPN-SAF containing the 10 most symptomatic and pertinent items [14]. This tool allows for rapid administration in clinical and trial formats and has replaced the MPN-SAF in most settings. The survey has been successfully cross-validated against the EORTC QLQ-C30 and is available in a variety of languages including English, Italian, German, French, Mandarin, Arabic, Spanish, Dutch, Swedish, Portuguese, Japanese, Hebrew, and Czech.

## 3. Origins of Inflammation in MPN Patients

In healthy individuals, the inflammatory cascade is driven by a delicate interplay between cellular responses and neurohormonal stimulatory factors/cytokines. Dysregulation of this system is a hallmark feature of the MPNs. Although the initial inciting event has yet to be clearly elucidated, all MPN disorders arise from genetic defects within pluripotent stem cell populations that accumulate over the disease course. JAK2V617F, a member of the Janus kinase signal transduction pathway, was the first recognized mutation inherent to the MPN population (PV 96%, ET 50%, and MF 50%) [15]. The role the Janus kinase cascade (including* JAK2*,* JAK2*,* JAK3*, and TYK2) plays in the signaling of inflammatory cytokines is well documented and profound. As JAKs are essential to the signaling of surface growth factor receptors and cytokines bereft of intrinsic kinase activity, constitutive activation, as observed in JAK2V617F, induces unregulated signaling of STAT transcription factors with resultant cellular growth and propagation [16]. STAT3, in particular, is linked closely with cancer development via activation of immunomodulatory cytokines (IL-6, IL-10, and IL-17), growth factors (FGF, VEGF), and matrix metalloproteinases [17]. These products further induce positive autofeedback through the JAK/STAT pathway, perpetuating cellular malignant potential. In general, cytokines (interleukins, interferons, and soluble growth factors; definitions in Table 2) are important regulators of cellular processes, particularly those involving immunomodulatory activities, cellular growth angiogenesis, and migration [18]. Cytokine dysregulation is believed to be associated with other mutations observed within MPNs (IDH1/2, TET2) but requires additional investigation [19].

Chronic inflammation has been hypothesized to play a supportive role in oncogenesis given its promotion of genomic instability through DNA mutations and epigenetic changes, prevention of tumor immune surveillance, and encouragement of clonal evolution [17, 20, 21]. MPN cells (leukocytes, platelets) with inherent hypersensitivity to cytokines and or growth factors respond in a proliferative fashion with resultant production of more stimulatory factors. As chronic inflammatory conditions, MPN disorders revolve around a perpetual cycle of DNA damage, cellular remodeling, and subsequent fibrosis [22]. This process has been a topic of great interest, especially as it relates to the heterozygous clinical presentation of MPN patients.

## 4. Inflammation and MPN Symptom Development

The relationship between chronic inflammation and MPN symptom development has also been a topic of recent interest. An evaluation of abnormal cytokine expression within myelofibrosis determined that primary myelofibrosis (PMF) patients had significantly increased levels of IL-1B, IL-1RA, IL-2R, IL-6, IL-8, IL-10, IL-12, IL-13, and IL-15 and TNF-1, G-CSF, IFN-*α*, MIP-1*α*, MIP-1*β*, HGF, INF-*γ*-IP, and VEGF in addition to reduced IFN-*γ* levels [23]. IL-2R, IL-12, IL-15, and IP-10 were independently predictive of inferior survival. IL-2R and IL-12 were associated with transfusion needs and HGF, MIG, and IL-1RA were associated with marked splenomegaly.

Evaluation of the association between cytokines and MF symptoms was undertaken in 2013 through an ad hoc analysis of 309 MF patients during the blinded phase of the COMFORT-1 trial evaluating ruxolitinib against placebo [24]. Changing levels of five cytokines was significantly associated with change in the MPN-SAF TSS when controlled for arm, visit-by-arm interaction, age, sex, and body mass index (BMI). Cytokines included VCAM1, LEPTIN, TNFRII, TIMP1, and B2MICG. IL-8 appears to play a uniquely important role within MPNs. As a potent chemokine, it has previously been shown within other malignancies to promote angiogenesis, induce leukocyte chemotaxis/activation, and stimulate cellular reproduction. A recent study determined IL-8 to be associated with elevated levels of circulating blasts and the presence of constitutional symptoms [23]. In polycythemia vera, patients demonstrate increased levels of IL-1RA, IL-4, IL-5, IL-6, IL-7, IL-8, IL-10, IL-12, IL-13, IFN-*γ*, GM-CSF, MCP-1, MIP-1*α*, MIP-1*β*, HGF, IP-10, MIG, MCP-1, PDGF-BB, TNF-*α*, IFN-*γ*, and VEGF [25, 26]. Conversely, PV patients also demonstrate lower levels of EGF and RANTES. PV patients also had significantly elevated levels of IL-7, GM-CSF, MIP-1*α*, IP-10, MIG, eotaxin, IFN-*γ*, and VEGF in comparison to primary MF patients. On multivariate analysis, MIP-1*β* was shown to be associated with inferior survival. In addition, hemoglobin count correlated with IL-4 and MCP-1, hematocrit count correlated with TNF-*α* and MCP-1, lymphocyte count correlated with IL-6 and TNF-1, and JAK2V617F mutation status correlated with TNF-1 and PDGF-BB [26]. An analysis of ET patients determined this population to have elevated levels of IL-1B, IL-4, IL-6, IL-8, IL-10, IL-12, HGF, GM-CSF, IFN-*γ*, MCP-1, PDGF-BB, TNF-*α*, and VEGF. Interestingly, IL-4, IL-8, GM-CSF, IFN-*γ*, MCP-1, PDGF-BB, and VEGF appeared to be significantly higher in ET patients when compared to PV populations and may serve as useful markers to distinguish the two disorders [26]. Also within ET patients, polynuclear cell counts were found to correlate with HGF, IL-6, IL-12, MG-CSF, and VEGF whereas red cell counts correlated with PDGF-BB levels. JAK2V617F positive status also correlated with PDGF-BB and TNF-*α* [26]. In comparing PV and ET patients with vascular complications versus those without complications, no significant differences in cytokine levels were noted. However, in comparing PV and ET patients with a history of vascular events, ET patients have significantly increased levels of IL-4, IL-8, GM-CSF, IFN-*γ*, MCP-1, and VEGF [26].

Interestingly, the specific combination of inflammatory markers appears to be as important as the type of factor present. In MF, the combination of TNF-*α* and TIMP-1 has been shown to promote survival of CD34+ stem cells whereas the combination of ATP and TNF-*α* has been shown to reduce proliferation [27]. Specific inflammatory markers are also associated with disease severity and complications. For example, pentraxin and CRP are well established to play a role in thrombosis and atherogenesis. These biomarkers have been associated with the development of major thrombotic events in PV and ET [28]. A recent study also identified low levels of IL-12 in MPN patients with vascular complications [26]. Altered levels of PDGF, FGF, and VEGFb have also been noted in stromal cells of patients with PV, ET, and PMF suggesting proinflammatory cytokines promote bone marrow fibrosis which is well established to contribute to anemia and subsequently fatigue [29, 30]. Cytokines (BMP-1, BMP-R2, BMP-6, and BMP-7) may have a role in promoting the advancement of MPNs from early to later stages [31]. Of special interest, the presence of specific gene mutations impacts the type and degree of cytokine expression. For instance, JAK2V617F positive patients have significantly higher levels of IL1B, IL-8, IL-17A, and IFN versus triple-negative (*JAK2*,* MPL* negative) patients [32]. Much has yet to be learned about the role cytokines play in MPN symptom development. The growing availability of cost-sensitive cytokine profile testing has offered us what can best be recognized as preliminary data on this complex topic. Below we discuss the available literature on specific MPN symptoms and their relationships to inflammation (Table 3).

### 4.1. Fatigue

Fatigue is a common complaint among cancer patients, present within 30–60% of the cancer population [33]. The symptom is particularly prominent within MPNs (PV 85%, ET 72%, and MF 84%), representing the most common symptom voiced regardless of subtype. MPN-fatigue has been shown to correlate closely with functional capacities. In a novel evaluation of MPN patient functionality, participants were found to perform an average of 25.1 metabolic equivalents (METS), akin to scores observed in Parkinson disease patients and dramatically lower than healthy controls (45.8 METS) [9].

Cytokines have been documented to induce fatigue in both malignant and nonmalignant states. A recent study identified positive associations between TNF-*α* and postchemotherapy fatigue in women with breast cancer [34]. Anemia is a recognized contributor to this symptom and recent studies have demonstrated that cytokines play a critical role in the development and perpetuation of this comorbidity. IL-1, IL-6, and TNF were recently shown to promote deregulation of erythropoietin with resultant anemia in acute myelogenous leukemia (AML) and myelodysplastic syndromes (MDS) [35, 36]. Cytokine-induced hypocortisolism is also a potential source of fatigue. Cytokines have been shown to induce dysregulation of the HPA axis and promote a blunted stress response due to subtherapeutic cortisol production [37]. In cancer, fatigue has been closely associated with depressed mood and increased levels of IL-6, a cytokine observed within MPNs [38, 39]. A survey of 1788 MPN patients confirmed that 32% have been seen or diagnosed with depression and 22.2% had received active treatment of mood disorder within the prior six months suggesting potential association in this population [40].

The combination of cancer-related depression and fatigue also contributes to a sedentary lifestyle which further encourages a proinflammatory state that propagates symptoms. Multiple randomized controlled trials have demonstrated that cancer-related fatigue may be reduced through aerobic physical activity, potentially through modulation of cytokine production [37]. The mechanism has yet to be elucidated as intense physical activity has been shown to increase circulating levels of IL-6 which subsequently stimulates the production of other anti-inflammatory cytokines including IL-1RA and IL-10, and inhibit proinflammatory cytokines such as TNF-*α*. The MPN Fatigue Project is an international effort performed in collaboration with the* MPN Forum* aiming at evaluating the breadth and efficacy of current strategies targeting MPN fatigue [41]. The study remains ongoing and includes evaluation of treatments such as sleep deprivation, dietary supplements, and exercise.

### 4.2. Splenomegaly

Abdominal-related complaints are common among MPN patients, largely attributable to splenomegaly, portal hypertension, mechanical obstruction, and splenic infarcts. An independent source of morbidity and mortality complaints related to the abdomen has included early satiety (76%), abdominal pain (63%), abdominal discomfort (72%), and weight loss (48%) [3]. However, cytokines may also play an important role in the development of this symptom. Splenomegaly has been associated with expansion of the malignant clone from the bone marrow microenvironment to extramedullary sites including the spleen. TNF-*α*, in particular, promotes clonal expansion in JAK2V617F positive MPNs [42, 43]. The development of splenomegaly has also been associated with specific cytokines including MIG, HGF, and IL-1RA [23]. However, the mechanism involved in stimulation has yet to be established. Interestingly, JAK2/STAT3 signaling has been shown to promote fibrosis, angiogenesis, and inflammation in the setting of portal hypertension, independent of the presence of malignancy suggesting that regional inflammation also plays a role in abdominal pain, whether or not cancer is present [44]. Thrombosis may result in a variety of abdominal complaints, particularly with ET and PV. An evaluation of 244 consecutive PV and ET patients demonstrated that patients within the highest CRP protein tertile had the highest rate of major thrombotic events [28]. Similarly patients demonstrating the lowest pentraxin 3 levels had higher risks for major thrombotic events. Of interest, values of hs-CRP and PTX3 also correlated with JAK2V617F allele burden.

Abdominal pain may also be exacerbated by cytokine-induced nerve hyperstimulation, both peripherally and centrally. Animal studies have shown increased expression of TNF-*α*, IL-1, and IL-6 after nerve injury, cytokines all disproportionately high in the MPN population. In addition, inflammation and trauma have been shown to induce peripheral nerve cell release of inflammatory cytokines within the central nervous system via glial stimulation [45]. The effects of cytokine-mediated pain were demonstrated in a recent study of patients with painful neuropathies where it was observed that this population had twofold higher levels of IL-2 mRNA and TNF-*α* mRNA in comparison to healthy controls [46]. The direct impact these cytokines have on nerves within MPN populations has yet to be investigated.

### 4.3. Microvascular, Cognitive Symptoms and Pruritus

Microvascular complaints typically refer to those symptoms that result from disease activity occurring at a capillary level. In the MPNs, these symptoms may include headaches, concentration problems, lightheadedness, dizziness, vertigo, numbness/tingling, and sexual dysfunction. Historically, neurocognitive disturbances have been attributed to cellular stasis and microthrombosis. Proinflammatory cytokine production is believed to be a contributor to cognitive impairment in cancer patients via the disruption of neurohormonal signaling and impaired creation of neurotransmitters. These neurotransmitters include serotonin, dopamine, and norepinephrine, all of which are critical to functions involving homeostasis of sleep, mood, and memory [47–49]. A recent analysis of patients treated with ruxolitinib in the COMFORT-II trials identified RANTES and Pal1 levels to correlate with the complaints of insomnia [50].

The role of inflammation in cognitive impairment has been intensely studied in both animal and human models. In an evaluation of IL-6 deficient animals, injection of lipopolysaccharide (LPS, shown to inhibit memory and learning in animals) failed to induce cognitive impairments suggesting IL-6 plays a key role in interrupting the process of memory and learning [49]. In human studies, elevated levels of IL-1, TNF-*α*, IL-6, and CRP were also linked to impaired memory and neurodegenerative disorders in the elderly [51–53]. Within hematological disorders, patients with elevated levels of IL-6 were found to have worsened executive function [36]. Interestingly, AML and MDS patients with higher levels of IL-8 were found to have improved memory. Pruritus has also been linked to the inflammatory cascade. A recent study of JAK2V617F transgenic mice demonstrated increased number of mast cells in those with the PV phenotype [54]. These mast cells represent a key source of prostaglandin, leukotriene, histamine, and tryptase, mediators of the inflammatory response with recognized ties to pruritus. A study evaluating symptoms of ruxolitinib treated patients within the COMFORT-II trial determined that baseline pruritus was associated with lower ferritin levels, a surrogate marker of inflammation [50]. Pruritus, most prominent in PV patients (65%), may be tied to an inflammatory cellular response as well. Basophils have been instigated as a primary mediator for symptom development and studies have demonstrated that the number of constitutively activated and hypersensitive circulating basophils is increased in PV, correlating with the degree of pruritus [55]. In addition, mast cells may play a role. Recent studies using infrared thermography have documented mast cell degranulation due to temperature shifts with the release of pyrogenic factors such as interleukins, histamine, and leukotrienes [56, 57].

### 4.4. Constitutional Symptoms

Fevers, night sweats, and weight loss are foundational symptoms in MPNs. Both fevers and night sweats are recognized to be partially cytokine driven, typically by IL-1, IL-2, IL-6, TNF-*α*, and IFN. Within other malignancies, IL-6 has been found to correlate with the presence of B symptoms which serve as a prognostic factor in Chronic Lymphocytic Leukemia (CLL) and Hodgkin's lymphoma [58–60].

The development of MPN-associated weight loss is complex and relates to a variety of factors including splenomegaly, portal hypertension, and cancer-cachexia. The role of a cytokine-driven proinflammatory state as the nidus for cancer-cachexia is well supported by literature [61, 62]. Defined as a process of dysregulated carbohydrate and fat metabolism with ongoing skeletal muscle breakdown, the presence of cancer-cachexia is linked to a dismal survival rate in comparison to cancer patients lacking this feature [63]. In cancer patients, TNF-*α* has been shown to induce proteolysis of skeletal muscle and furthermore enhance the expression of genes related to enzymes in the ubiquitin-dependent proteolytic pathway [64]. In a review of cytokine levels present in patients from the COMFORT-II trial, weight loss was associated with lower leptin levels and high CD40L was associated with loss of appetite. Whether these aberrant cytokines function to support cancer-cachexia or involve an alternative mechanism of action has yet to be investigated.

## 5. Improving Symptoms by Targeting Cytokines

Recognizing the substantial impact chronic inflammation has on the MPN symptom burden, attentions have turned to therapies demonstrating efficacy in reducing cytokines. As in other malignancies, improving physical activity and reducing fat intake may reduce inflammatory cytokines and improve survival [65]. In noncancer patients, increased physical activity has been shown to reduce TLR4 signaling and truncate release of inflammatory cytokines [66]. In obese subjects, proinflammatory cytokines have also been shown to be released by white fat which may be subsequently removed through physical activity. In addition to evaluating the effects of sedentary living on the MPN symptom burden, the final phases of the MPN Fatigue Project involve the development of comprehensive patient activity programs which may have subsequent impact on cytokine-induced symptomatology [41].

Recognizing the role constitutive Janus kinase signaling plays in inflammation,* JAK2* inhibition has become a rational target for preventing cytokine dysregulation. A recent study evaluated meaningful changes in cytokine expression following 24 months of ruxolitinib therapy in 63 high-risk MF patients [67]. Ruxolitinib was able to induce profound reductions in the expression of TNF-*α* and MIP-1*α* at both 4 weeks and 24 months. The expression of IgE was also strongly reduced in almost all patients with direct impact on the amount of activated anti-inflammatory macrophages. A similar study utilized the Functional Assessment of Cancer Therapy-Lymphoma (FACT-Lym) to evaluate symptoms of ruxolitinib treated patients within the COMFORT II trial and compare them to changes in cytokine levels [50]. Ten symptoms including fever, weight loss, fatigue, loss of appetite, pain, itching, sleeping well, lack of energy, night sweats, and trouble sleeping were assessed at baseline and weeks 8, 24, and 48. Treatment with ruxolitinib led to improved items of itching, night sweats, and weight loss with subsequent reduction in numerous cytokines. Loss of appetite improved over time and negatively correlated with decreases in IL-1RA levels in ruxolitinib treated patients.

The impact emerging* JAK2* inhibitors such as momelotinib and pacritinib will have on cytokines is of high interest as they progress through clinical trials. Importantly, their limited hematological toxicities may be reflective of their selective inhibition of kinases. A recent investigation of pacritinib demonstrated that it selectively spares* JAK1* while inhibiting* JAK2*, JAK2V617F, FLT3, and IRAK1, an IL-1 receptor kinase associated with the inflammatory response and suppression of normal hematopoiesis [68]. Whether inhibition of IRAK1 is of clinical significance from a symptomatic standpoint has yet to be investigated. Other cellular signaling networks such as the PI3K-Akt-mTOR pathway impact cytokine development and represent novel targets for intervention. Similarly, as antifibrosing agents, hedgehog inhibitors, hypomethylating agents, histone deacetylation inhibitors, and HSP90 inhibitors enter the treatment landscape, knowledge of their impact on inflammation is of great interest.

## 6. Conclusion

Clear relationships exist between MPN symptoms and markers of inflammation. Though most data remains in early stages of investigation, knowledge gleaned from other malignancies has offered us potential mechanisms that explain these observed cytokine-symptom associations. A variety of MPN symptoms correlate with the presence of specific markers of inflammation. However, how these markers differ between MPN subtypes, change with disease progression, and relate to transformation remain unknown. The expanded access to targeted agents has provided a platform by which cytokine signals may be inhibited early within the cascade, limiting their potential for toxicity. It is with great anticipation that we venture into this uncharted territory of cytokine-symptom associations and explore novel therapies hosting high potential for symptomatic benefit.

## Figures and Tables

**Table 1:** MPN symptom assessment forms.

PRO tool	Year	Total number of questions	Question composition	Available languages
MF-SAF [9]	2009	20	Fatigue Inactivity Bone pain Cough Pruritus Night sweats Fever Weight loss Abdominal pain/discomfort Early satiety Quality of life	English

MPN-SAF [3]	2011	27	Fatigue Inactivity Headache Dizziness Concentration problems Numbness Insomnia Sad mood Sexuality problems Bone pain Cough Pruritus Night sweats Fever Weight loss Abdominal pain/discomfort Early satiety Quality of life	English Italian German French Mandarin Arabic Spanish Dutch Swedish Portuguese Japanese Hebrew Czech

MPN-SAF TSS [14]	2013	10	Fatigue Inactivity Concentration problems Bone pain Pruritus Night sweats Fever Weight loss Abdominal discomfort Early satiety	English Italian German French Mandarin Arabic Spanish Dutch Swedish Portuguese Japanese Hebrew Czech

MF-SAF: Myelofibrosis Symptom Assessment Form; MPN-SAF: Myeloproliferative Neoplasm Symptom Assessment Form; MPN-SAF TSS: Myelofibrosis Symptom Assessment Form Total Symptom Score; PRO: Patient Reported Outcome; QOL: quality of life.

**Table 2:** Cytokine descriptions.

Acronym	Description
B2MICG	Beta-2 microglobulin
BMP6	Bone morphogenetic protein 6
CRP	C-reactive protein
FGF	Fibroblast growth-factor
GCSF	Growth colony stimulating factor
HGF	Hepatocyte growth factor
Hs-CRP	High-sensitivity C-reactive protein
IFN-*α*	Interferon-alpha
IFN-*γ*	Interferon-gamma
IFN-*γ*-IP	Interferon-gamma inducible protein
IP-10	Interferon-gamma inducible protein 10
IL-1B	Interleukin-1B
IL-1RA	Interleukin-1RA
IL-2R	Interleukin-2R
IL-6	Interleukin-6
IL-8	Interleukin-8
IL-10	Interleukin-10
IL-12	Interleukin-12
IL-13	Interleukin-13
IL-15	Interleukin-15
IL-17	Interleukin-17
MIG	Monokine-induced by gamma
MIP-1*β*	Macrophage inflammatory protein-1*β*
NF-KB	Nuclear factor-KB
PAI1	Plasminogen activator inhibitor-1
PTX3	Pentraxin-3
TIMP1	Tissue inhibitor of metalloproteinase-1
TNF-1	Tumor necrosis factor-1
TNF-*α*	Tumor necrosis-factor-*α*
TNF-RII	Tumor necrosis-factor-RII
VCAM1	Vascular adhesion molecule
VEGF	Vascular endothelial growth factor
EGF	Epidermal growth factor

**Table 3:** Associations between MPNs and cytokines.

Inflammatory marker^*∗*^	Impact	Disorder
B2MICG	Symptoms	MF
BMP1	Disease advancement	PMF
BMP6	Disease advancement	PMF
BMP7	Disease advancement	PMF
BMP-Rcp2	Disease advancement	PMF
CD40L	Loss of appetite	MF
CRP	Thrombosis; atherogenesis	PV, ET
Ferritin	Pruritus	MF
FGF	Marrow fibrosis	PV, ET, PMF
HGF	Splenomegaly	PMF
IFN	Associated with JAK2V617F	MF
IL-12	Inferior survival; transfusion requirements, vascular complications	MF
IL-15	Inferior survival	MF
IL-17A	Associated with JAK2V617F	MF
IL-1B	Associated with JAK2V617F	MF
IL-1RA	Splenomegaly	PMF
IL-2R	Inferior survival; transfusion requirements	MF
IL-8	Elevated blasts; constitutional symptoms	MF
IL-8	Associated with JAK2V617F	MF
IP-10	Inferior survival	MF
LEPTIN	Symptoms; weight loss	MF
MIG	Splenomegaly	PMF
PAL1	Insomnia	MF
PTX	Thrombosis; atherogenesis	PV, ET
RANTES	Insomnia	MF
TIMP1	Symptoms	MF
TNF-1	Clonal expansion	JAK2V617F+ MPNs
TNFRII	Symptoms	MF
VCAM1	Symptoms	MF
VEGFb	Marrow fibrosis	PV, ET, PMF

ET: essential thrombocythemia; MF: myelofibrosis; MPN: myeloproliferative neoplasm; PMF: primary myelofibrosis; PV: primary myelofibrosis.

^*∗*^Refer to Table 2 for definition.
